# Performance and mechanism analysis of DMP-30 modulated waterborne epoxy resin emulsified asphalt

**DOI:** 10.1371/journal.pone.0347688

**Published:** 2026-05-07

**Authors:** Jiao Yan, Li Chai, Le Zhang, Xiaoyu Wang

**Affiliations:** 1 School of Aeronautical Manufacturing and Vehicle Engineering, Xihang University, Xi'an, Shaanxi, China; 2 The Key Laboratory of Intelligent Construction and Maintenance of CAAC, Chang'an University, Xi'an, China; 3 Inner Mongolia Research Institute of Transportation Science Development, Hohhot, China; 4 Comprehensive Support Center of Inner Mongolia Autonomous Region Department of Transportation, Hohhot, China; Shandong University of Technology, CHINA

## Abstract

The rapid breakage of emulsified asphalt can hinder the curing process of epoxy resin, thereby compromising the performance of waterborne epoxy resin emulsified asphalt (WEREA) and its mixture. Introducing the DMP-30 curing accelerator to expedite the curing of waterborne epoxy resins (WER) presents a possible solution. This study aims to investigate the impact of varying dosages of DMP-30 (0%, 1%, 2%, and 3%) on the performance of WEREA and its mixes through macro- and micro-experimentation. A series of experiments was used to evaluate the high-temperature properties, adhesion properties, microscopic properties, and abrasion resistance of the samples. These experiments included temperature scanning tests, pull-out tests, Fourier transform infrared spectroscopy (FTIR) tests, and abrasion tests, etc. The macro test results revealed both positive and negative effects of DMP-30 on the performance of the binder and mixture. The most significant positive enhancement was observed at a 1% dosage of DMP-30, resulting in the highest rutting factor and adhesion strength of binder, as well as the greatest dynamic stability of the mixture. Furthermore, the freeze-thaw and immersion abrasion values were minimized at this dosage. FTIR tests demonstrated that a moderate amount (1%) of DMP-30 facilitated the reaction between the curing agent and the epoxy resin, while an excessive amount (3%) interfered with the curing reaction, leading to residual curing agent. Additionally, abrasion test results indicated that the addition of an appropriate amount (1%) of DMP-30 at a higher curing temperature (35 °C) reduced the abrasion loss within a shorter timeframe.

## 1. Introduction

Micro-surface treatment is a preventive measure widely used in asphalt pavement maintenance [1,2]. However, owing to the difference in binder properties, treated micro-surfaces are highly susceptible to loose aggregate and rutting early in service [3]. Waterborne epoxy resins (WER), as thermosetting materials with strong adhesive properties, have attracted increasing attention from researchers in the field of road materials [4].

Presently, the performance of waterborne epoxy resin emulsified asphalt (WEREA) has been predominantly studied from two perspectives: macroscopic and microscopic [5–11]. For example, LI et al. explored the performance of WEREA through tensile strength and linear amplitude scan tests, revealing that a 5% epoxy resin content significantly enhanced the bond strength and fatigue life of emulsified asphalt [12], while advanced microscopic characterization methods have provided insights into asphalt molecular behavior [10]. LIU et al. compared and analyzed the properties of WEREA and SBR-modified emulsified asphalt, concluding that the adhesion performance of WEREA was superior when the WER content exceeded 11% [13–15]. ZHANG et al. observed that the high-temperature performance of emulsified asphalt increased with higher WER admixture, yet the storage stability and low-temperature performance decreased [16,17]. Additionally, they found that the WER underwent both physical and chemical reactions with the curing agent, subsequently cross-linking with the asphalt to form a three-dimensional spatial network structure, thereby exhibiting excellent high-temperature performance and waterproof performance [18]. GU et al. delved into the formation mechanism of WEREA strength using fluorescence microscopy and scanning electron microscopy, identifying the formation of an “island” structure by the epoxy resin and asphalt as a crucial factor [19,20]. CHEN et al. pointed out that in the case of two-emulsion WER, the curing rate and final strength of WER were controlled by the diffusion of curing agent into the epoxy resin [21]. Lv et al. demonstrated that the rapid demulsification of emulsified asphalt would restrict the physical fusion and chemical reaction of epoxy and curing agent, consequently limiting the strength formation of WEREA [22,23].

The incorporation of WER can significantly enhance the adhesion performance of emulsified asphalt, making it an ideal material for micro-surface treatment [23,24]. HAN et al. determined the mix ratios of two aggregate micro-surface mixtures through Wet Track Abrasion Tests (WTAT) and wheel load tests, finding that the optimal slip resistance was achieved at a basalt micro-surface containing 12% WER admixture and a 7.4% to 8.4% oil-to-stone ratio [23,25]. XU et al. observed that WER substantially improved the wear resistance and water damage resistance of the mixture at the micro-surface, while also reducing low-temperature performance, as evidenced by 1-hour wet track abrasion tests and loaded wheel tests [24]. JI et al. demonstrated favorable waterproof, anti-skid, and anti-rutting properties of WEREA through tests on British Pendulum Number (BPN), water penetration coefficient, wheel rutting deformation rate, and rutting dynamic stability [26]. ZHENG et al. found that the incorporation of WER enhanced the freeze-thaw stability (refers to the resistance to damage after repeated freezing and thawing cycles, measured by freeze-thaw WTAT) and water stability of the micro-surface by performing a wet wheel abrasion test on the WER surface after water immersion and freeze-thaw cycles [27]. In summary, extensive research has been conducted on the performance of WEREA and its cold mixture [23–27]. Nevertheless, with the development of intelligent construction technologies [28–30] and advanced material characterization methods [10,11,31], further investigation is needed to optimize WEREA performance.

While various curing accelerators have been explored to address this issue, the selection of an optimal accelerator that can precisely match the demulsification rate of WEREA and enhance its performance at practical construction temperatures remains a research gap. To bridge this gap, this study introduces 2,4,6-Tris(Dimethylaminomethyl)phenol (DMP-30), a tertiary amine-based curing accelerator, and investigates its unique potential in regulating the curing process of WEREA. Unlike some conventional accelerators that may cause excessive reactivity or adversely affect the emulsion stability, DMP-30 is known for its efficiency at relatively low dosages and its ability to promote curing without significantly disrupting the emulsion. This study aims to systematically evaluate the impact of varying dosages of DMP-30 (0%, 1%, 2%, and 3%) on the performance of WEREA and its mixes through macro- and micro-experimentation. The findings are expected to provide valuable insights for optimizing WEREA formulations and addressing the curing-demulsification mismatch, thereby improving the reliability and performance of micro-surface treatments.

## 2. Materials and methods

### 2.1. Materials

#### 2.1.1. Emulsified asphalt.

The emulsified asphalt used in this study was prepared using PUMA 70# base asphalt and BE-3X slow-cracking and fast-setting cationic emulsifier provided by Xi'an Starlight Energy Co., Ltd (China). The main indicators of the emulsified asphalt are presented in Table 1. The test method is based on JTG E20-2011 specification (China), and all the results complied with the requirements of JTG 5142−2019 (China).

**Table 1:** BE-3X emulsified asphalt main indicators.

Item		Unit	Result	Requirement
Engler Viscosity (E_25_)		s (or °E)	21.0	3-30
Evaporation residue content		%	61.3	≥60
Residue performance	Penetration (100g, 25℃, 5s)	0.1 mm	82.5	40-100
	Softening point	℃	55.0	≥53
	Ductility (5°C)	cm	23.0	≥20

#### 2.1.2. Waterborne epoxy resin, curing agent, and accelerator.

In this study, the waterborne epoxy resin (WER) and curing agent (Triethylenetetramine, TETA) provided by Nanjing University of Technology (China) were utilized. The main indicators of waterborne epoxy resin and curing agent are presented in Table 2. Based on preliminary experiments and previous research [15,23,25], the mass ratio of the curing agent to the WER was determined to be 1:1 (by solid content) based on the epoxy value of the WER and the amine value of the curing agent, aiming to achieve a near-stoichiometric balance for the epoxy-amine curing reaction. This means the mass of solids in the curing agent accounts for 50% of the mass of solids in the WER, which is crucial for ensuring complete curing and forming a dense network structure. Furthermore, the solid content of the WER was optimized to range from 0% to 20% of the mass of asphalt in the emulsified asphalt. This range was selected based on experimental findings that while WER significantly improves high-temperature stability and adhesion strength, its thermosetting nature leads to increased brittleness and reduced low-temperature performance.

**Table 2:** Main indicators of waterborne epoxy resin and curing agent.

Item	Unit	Water-based epoxy resin	Curing agent
Appearance	–	Milky white liquid	Clear color liquid
Solid content	%	50 ± 2	50
Epoxy value	mol/ 100g	0.2319	0.0059
Amine value	mol/ 100g	–	56.22

In order to accelerate the curing rate of WER at normal and low temperatures, DMP-30 curing accelerators provided by Xi'an Huaze Road Materials Co., Ltd (China) were utilized to modulate the performance of WEREA and its mixes. The dosage of DMP-30 was maintained within 0%−3% of the mass of epoxy resin [1,23]. Preliminary tests indicated that dosages below 1% showed minimal acceleration effect, while dosages above 3% led to adverse effects on the emulsion stability and final performance, such as reduced adhesion and increased brittleness. Therefore, the 0%−3% range was determined to be the optimal window for investigating the performance regulation of DMP-30. The key indicators are presented in Table 3.

**Table 3:** DMP-30 curing accelerator main indicators.

Item	Unit	Result
Main components	–	2,4,6-Tris (Dimethylaminomethyl) phenol
Appearance	–	Yellow transparent liquid
Amine value	mol/ 100g	580-630
Viscosity	mPa·s	100-260

#### 2.1.3. Aggregate, filler, and water.

In this study, the basalt aggregates utilized were sourced from a construction site in Xi'an, China. The main indicators of these aggregates are presented in Table 4. The fillers employed comprised P.O 42.5 silicate cement and limestone ore powder, while ordinary drinking water was used as the solvent. The test method is based on JTG E20-2011 (China), with all results meeting the requirements stipulated by JTG 5142−2019 (China).

**Table 4:** Main indicators of basaltic aggregate.

Item		Unit	Result	Requirement
Coarse Aggregate	Crushing value	%	22.0	≤26
	Needle flake content	%	11.7	≤15
	Robustness	%	5.8	≤12
Fine Aggregate	Sand equivalent	%	71.0	≥65
	Robustness	%	10.0	≤12

### 2.2. Waterborne epoxy resin emulsified asphalt preparation

**Fig 1:**
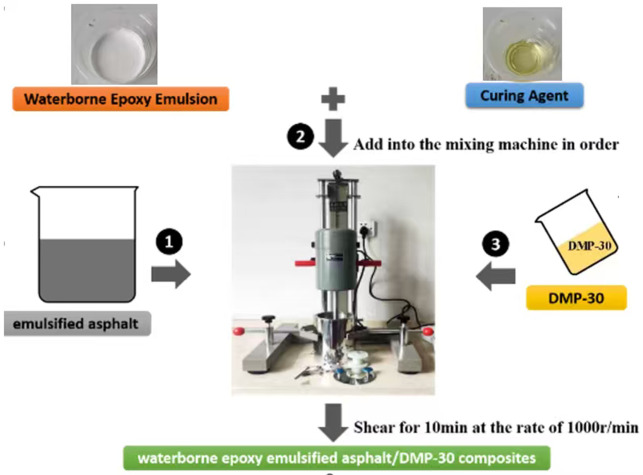
Water-based epoxy resin emulsified asphalt preparation process.

According to previous studies and preliminary experiments [1,32–34], the process of preparing waterborne epoxy emulsified asphalt (WEREA) is illustrated in Fig 1. Firstly, WER (0%−20%) and curing agent were mixed at a ratio of 1:0.5 and added to the emulsified asphalt. Then, DMP-30 curing accelerator (0%−3%) was blended with the emulsion asphalt system. Finally, the WEREA was obtained by thoroughly mixing using a high-speed shear mixer (1000 r/min for 10 minutes).

### 2.3. Waterborne epoxy resin emulsified asphalt micro-surface mixture

A well-designed gradation is advantageous for enhancing the performance of the mixture. The MS-3 gradation type offers specific anti-slip properties, while the inclusion of coarse aggregate provides both strength and a broader range of applications. In this study, the MS-3 gradation was selected for micro-surface design, with its synthetic gradation presented in Table 5. Based on preliminary experiments and previous research findings [23,25], an oil-to-stone ratio of 6.8% was determined.

**Table 5:** Synthetic gradation of aggregate for micro-surfacing mixture.

Aggregate specification	Sieve size (mm)							
	9.5	4.75	2.36	1.18	0.6	0.3	0.15	0.075
5-10 (mm)	20.0	3.1	1.0	0.2	0.2	0.2	0.2	0.2
3-5 (mm)	20.0	20.0	18.2	2.8	0.8	0.4	0.3	0.3
0-3 (mm)	50.0	50.0	40.5	28.0	18.3	9.3	4.4	1.4
Mineral powder	10.0	10.0	10.0	10.0	10.0	10.0	9.3	7.8
Synthetic gradation (%)	100.0	81.4	54.3	39.0	28.9	19.7	14.2	9.6

### 2.4. Experimental program

**Fig 2:**
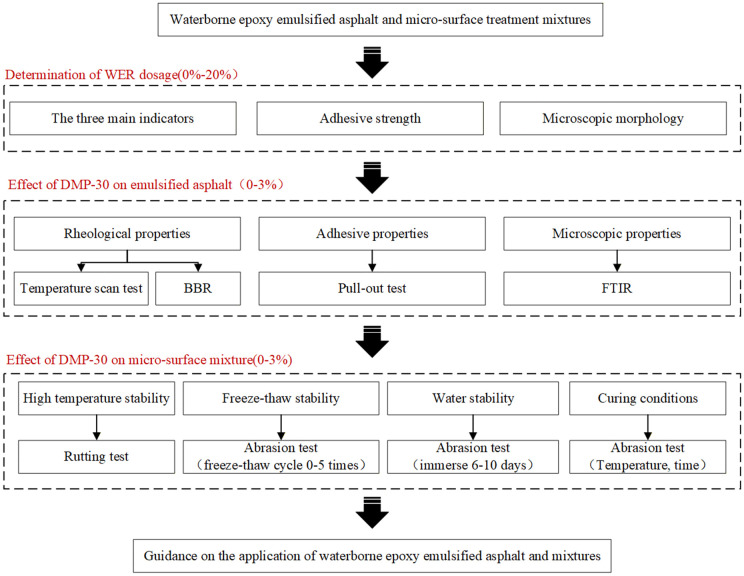
Flow chart of the experimental program.

Following the objectives outlined above, the experimental program was designed to systematically evaluate the performance of WEREA and its mixes. The research plan is shown in Fig 2.

The experimental program was designed to investigate the impact of varying dosages of DMP-30 (0%, 1%, 2%, and 3%) on the performance of WEREA and its mixtures through integrated macroscopic and microscopic analyses (Fig 2). Initial characterization focused on fundamental physical properties of WEREA, including needle penetration and adhesive strength, complemented by fluorescence microscopy to determine the optimal WER dosage. Subsequent evaluation examined the effects of DMP-30 on high-temperature, low-temperature, and adhesion properties through temperature scanning tests, bending beam rheology tests, and pull-out tests. Microscopic insights were provided through Fourier transform infrared (FTIR) spectroscopy to elucidate the regulatory mechanisms of DMP-30. Mixture performance was assessed using rutting tests and abrasion tests under various conditions (freeze-thaw and water immersion treatments) to examine the influence of DMP-30 on high-temperature stability, freeze-thaw stability, and water stability. Finally, based on abrasion test results, the effects of DMP-30 dosage and maintenance temperature on strength formation in WEREA mixtures were analyzed to establish a foundation for practical applications. All tests were conducted with three replicates (n = 3) unless otherwise specified. Results are presented as mean ± standard deviation (SD).

The macroscopic tests (e.g., rutting and abrasion tests) directly evaluate the overall performance of the WEREA mixes, while the microscopic tests (e.g., FTIR) provide insights into the underlying chemical reaction mechanisms. By linking the observed macroscopic properties to their microscopic origins, these tests complement each other and thus validate the research hypothesis.

#### 2.4.1. Temperature scan test.

The temperature scanning tests on WEREA with various DMP-30 dosages were conducted using a dynamic shear rheometer (TA Discovery Series HR-3, TA Instruments Waters, Delaware, USA) in accordance with AASHTO T315-12. The test temperature was set between 52°C and 82°C, while the frequency was set to 10 rad/s. Three replicates were tested for each DMP-30 dosage.

#### 2.4.2. Bending beam rheological test.

The present study conducted bending beam rheology (BBR) tests on WEREA with varying dosages of DMP-30 using a bending beam rheometer (Cannon TE-BBR SD, CANNON Instrument Company, State College, Pennsylvania, USA), following the guidelines of AASHTO TP 122−16. The test temperatures were set at −6°C, −12°C, and −18°C. Each temperature condition was tested with three replicates.

#### 2.4.3. Pull-out test.

In this paper, with reference to GB/T 5210, a pull-out test was conducted on WEREA with various DMP-30 dosages by using an adhesion tester (XH-M type, Tiandi Starfire Technology Development Ltd, Beijing). And the spindle size is 20 mm. Five replicates were tested for each DMP-30 dosage to ensure statistical reliability given the higher variability inherent in adhesion testing.

#### 2.4.4. Fourier transform infrared spectroscopy test.

In order to analyze the effect of DMP-30 on the curing reaction of WEREA, Fourier Transform Infrared Spectroscopy tests (FTIR) were performed on WER, DMP-30, WER + DMP-30, WEREA+DMP-30 (0%), WEREA+DMP-30 (1%), WER + DMP-30 (2%), and WEREA+DMP-30 (3%) in this paper. The Nicolet IS 50/6700 FTIR spectrometer (Shanghai, China) was used for testing. Three replicate measurements were taken for each sample type.

#### 2.4.5. Rutting test.

**Fig 3:**
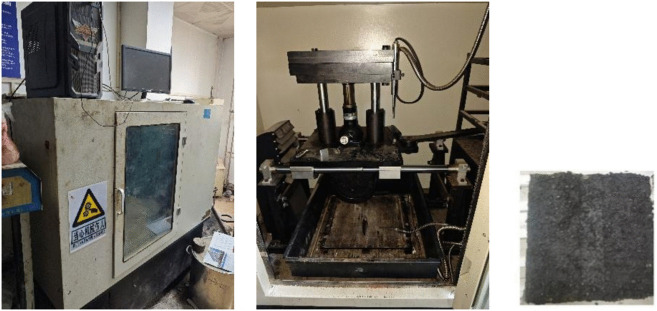
Automatic rutting tester and micro-surfacing rutting specimen.

The micro-surface can repair the rutting problem of the pavement, and it should have certain high-temperature stability itself. In this study, an AC-13 asphalt mixture rutting plate was used as the coating carrier of micro-surface, and its dynamic stability was tested according to the Chinese standard JTG-E20-2011 (T 0719-2011). The test was conducted using a fully automatic rutting tester (e.g., model HYCZ-5, as shown in Fig 3), which consists of a loaded wheel tracking system, a temperature-controlled chamber, and a data acquisition system.

The design thickness of the micro-surfacing overlay for rutting repair was set at 1 cm. An AC-13 asphalt mixture rutting slab was selected to serve as the substrate material for the water-based epoxy micro-surfacing. After cleaning the rutting slab, the prepared water-based epoxy emulsified asphalt micro-surfacing mixture was uniformly spread over it; the specimen was then cured in an oven at 60°C for a minimum of 16 hours before undergoing the rutting dynamic stability test. Three replicate specimens were tested for each DMP-30 dosage.

The rutting depth was automatically recorded by a linear variable differential transformer (LVDT) displacement sensor every cycle. The high-temperature stability of the micro-surface mixture is evaluated by the parameter Dynamic Stability (DS), which is defined as the number of load passes required to generate 1 mm of rutting deformation within a specific deformation interval (typically between 45 min and 60 min of the test). The DS value (times/mm) is calculated using the following formula.

$$DS=\frac{(t_{\text{2}}-t_{\text{1}})\times N}{d_{\text{2}}-d_{\text{1}}}\times C_{\text{1}}\times C_{\text{2}}$$

Where: DS = Dynamic stability (times/mm); $t_{\text{1}}$ = Time at the beginning of the deformation interval, typically 45 min;$t_{\text{2}}$ = Time at the end of the deformation interval, typically 60 min; $d_{\text{1}}$ = Rutting depth corresponding to time $t_{\text{1}}$ (mm); $d_{\text{2}}$ = Rutting depth corresponding to time $t_{\text{2}}$ (mm); N = Travel speed of the test wheel, typically 42 passes/min; $C_{\text{1}}$ = Correction factor for the test wheel type (for the standard solid rubber wheel specified in JTG E20-2011, $C_{\text{1}}=\text{1}.\text{0}$); $C_{\text{2}}$ = Correction factor for the specimen size (for standard 300 mm × 300 mm specimens, $C_{\text{2}}=\text{1}.\text{0}$).

#### 2.4.6. Abrasion test.

The thermosetting material WEREA exhibits increased low-temperature brittleness after curing, necessitating the need for freeze-thaw stability in the preparation of micro-surface samples. Additionally, it is crucial for the micro-surface to maintain excellent water stability. This paper refers to the specification JTG-E20-2011 (T 0752–2011) to conduct wet track abrasion tests at WEREA micro-surfaces treated with freeze-thaw cycles 1–5 times and water immersion for 6–10 days. Among them, (1) the samples were frozen in a refrigerator at −20°C for 16h and then kept in a water bath at 25°C for 8h for 1 freeze-thaw cycle. (2) Water immersion treatment is to place the samples in a 25°C water bath for a certain period of time. Further, in order to provide guidance on the construction of WEREA micro-surface mixtures at different temperatures, this paper uses dry track abrasion tests to study the molding process of the sample at temperatures of 15°C, 25°C and 35°C. Three replicate specimens were prepared and tested for each condition.

## 3. Results and discussions

### 3.1. Effect of WER dosage on the properties of emulsified asphalt

#### 3.1.1. Basic physical properties.

The three main indicator tests provide a more comprehensive characterization of the fundamental physical properties of waterborne epoxy resin emulsified asphalt (WEREA). Table 6 shows the test results of the three main indicators of WEREA with different WER dosages. As can be seen from the table, as the WER dosage amount increases from 0% to 20%, the needle penetration decreases by 75.8%, the softening point increases to unmeasurable, and the ductility decreases to 10 mm. The softening point at 20% WER dosage exceeded the upper measurement limit of the ring-and-ball apparatus (>150 °C) due to the excessive hardness of the sample, which had formed a rigid three-dimensional crosslinked network that prevented proper measurement. The sample was physically too rigid for the standard testing procedure.

**Table 6:** Effect of WER dosage on the three main indicators of waterborne epoxy resin emulsified asphalt.

WER dosage (%)	0	5	10	15	20
Needle penetration (mm)	82.5	65	46	37	20
Ductility (mm)	>100	77	42	12	10
Softening point (℃)	55.0	62	75	88	–

**Fig 4:**
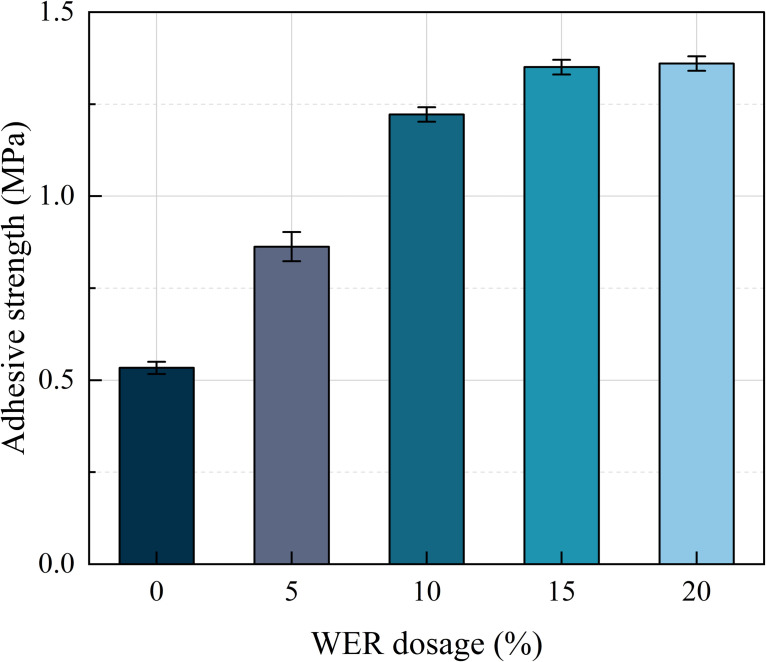
Variation of emulsified asphalt adhesive strength with waterborne epoxy resin dosage (mean ± SD, n = 5 for pull-out tests, n = 3 for others).

The binder, WEREA, should possess a specific adhesive strength to ensure adequate stability of the mixture and pavement structural layer. Fig 4 illustrates the variation in adhesive strength of WEREA with varying dosages of WER. From the figure, it can be seen that the increase of WER dosage from 0% to 20% increases the adhesive strength of WEREA by 62.0%, 130.1%, 154.3%, and 157.1% relative to the 0% WER dosage, respectively, and the increase shows a slowing down trend. The improvement of the adhesive strength is significantly reduced at WER dosage levels between 15% and 20%. This is due to the fact that the three-dimensional mesh structure in WEREA has been initially formed at a dosage level of 15%.

#### 3.1.2. Micro-morphological characteristics.

**Fig 5:**
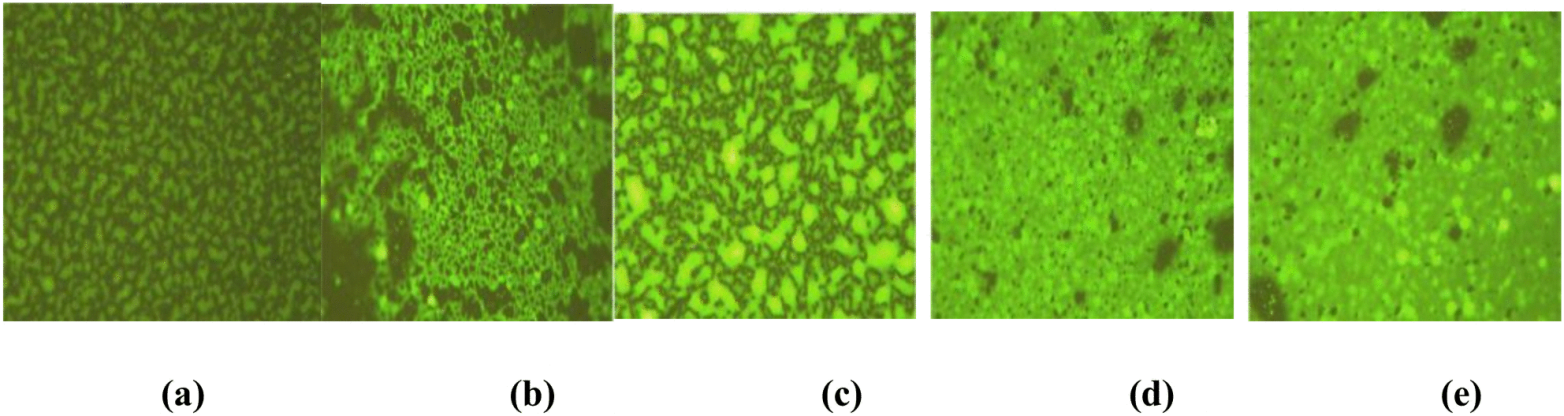
Fluorescence microscopy results of emulsified asphalt with various WER dosages. **(a)** WER (0%). **(b)** WER (5%). **(c)** WER (10%). **(d)** WER (15%). **(e)** WER (20%).

In order to investigate the strength molding mechanism of WEREA, fluorescence microscope smears of WEREA with varying levels of WER dosage were prepared using the method proposed by Martínez Anzures [35]. The samples were then left at 60°C for 6 hours. The test results are presented in Fig 5. From Figures (a), (b), and (c), it can be seen that the WER dosage increased from 0% to 10%, the epoxy phase gradually increased and aggregated into larger yellow-green spots, indicating the progressive development of a crosslinked mesh structure within the epoxy resin. Figure (d) reveals that at a WER dosage level of 15%, a more pronounced phase transition occurring in the WEREA system, where the epoxy resin phase becomes continuous while the asphalt transforms into dispersed phases. At this time, the WEREA system mainly shows the properties of the epoxy resin, indicating improved high-temperature stability and adhesion properties. Furthermore, when considering Figure (e), an increase in WER dosage does not significantly alter microscopic morphology, thereby providing microscopic evidence supporting Fig 4's observation regarding deceleration in adhesion property growth trend. To summarize from both economic and performance perspectives, the WER dosage of 15% was used for subsequent tests in this paper.

### 3.2. Effect of DMP-30 on waterborne epoxy emulsified asphalt

#### 3.2.1. High temperature rheological properties.

**Fig 6:**
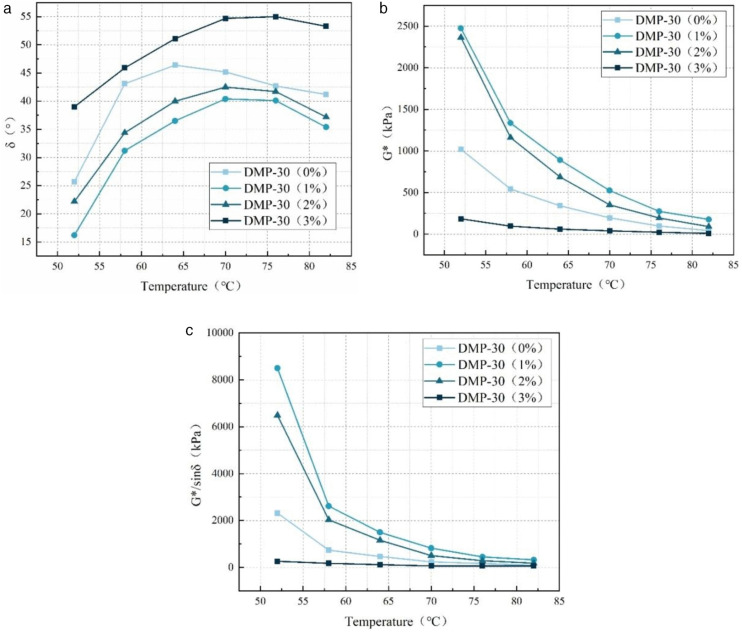
Results of WEREA temperature scanning tests at various DMP-30 dosages. **(a)** Phase angle; **(b)** Complex modulus; **(c)** Rutting factor.

The results of temperature scanning tests of WEREA at various DMP-30 dosages are presented in Fig 6. From the figure, with DMP-30 (1%) as the dividing line, an increase in DMP-30 dosage from 0% to 3% leads to a trend of initially increasing and then decreasing complex modulus and rutting factor values under the same temperature. Conversely, the phase angle exhibits an opposite behavior. This phenomenon can be attributed to the fact that an appropriate amount (1%−2%) of DMP-30 promotes the curing reaction of WEREA, resulting in a denser three-dimensional crosslinked mesh structure and enhanced resistance against deformation. Conversely, the performance decrease at higher dosages (e.g., 3% DMP-30) can be attributed to a kinetic imbalance caused by competitive reactions. While a moderate amount of DMP-30 catalyzes the reaction between TETA and epoxy groups, excessive tertiary amine groups tend to compete directly with TETA for epoxy groups. From the perspective of reaction kinetics, this competition leads to the formation of quaternary ammonium salts or initiates epoxy homopolymerization, which terminates chain growth or forms structural units that are incompatible with the primary cross-linking network. Consequently, this results in inhomogeneous network structures with lower crosslink density and residual curing agents, as further evidenced by the FTIR results, thereby compromising the mechanical performance.

In addition, when the dosage of DMP-30 is 1%, and the temperature rises from 52°C to 82°C, the G*/sin δ of the sample is 8498.2kPa, 2613.1kPa, 1497.5kPa, 821.8kPa, 448.1kPa, and 323.5kPa respectively. The values of the samples are all the largest, indicating that the modulation enhancement effect of DMP-30 is the best at this dosage, making the samples have strong high-temperature deformation resistance.

#### 3.2.2. Low temperature rheological properties.

**Fig 7:**
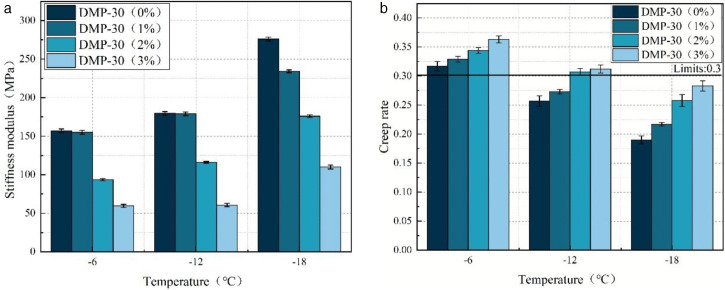
BBR test results of WEREA at various DMP-30 dosages. **(a)** Stiffness modulus. **(b)** Creep rate.

Fig 7 shows the BBR test results of WEREA under various DMP-30 dosages. With an increase in DMP-30 dosage at the same temperature, the stiffness modulus S of the sample demonstrates a decreasing trend, while the creep rate shows an increasing trend. This observation suggests that the addition of DMP-30 enhances the low-temperature deformation ability and stress relaxation performance of WEREA. This may be that the addition of DMP-30 results in a faster and more complete curing reaction between the curing agent and the WER, forming a more homogeneous sample [22,36]. When the dosage of DMP-30 increases from 0% to 3%, the stiffness modulus S of the samples at −6°C is less than 300MPa, and the creep rate is greater than 0.3, which meets the requirements of AASHTO specification. However, it should be noted that at −12°C and −18°C, the creep rates did not meet specifications, indicating limited enhancement of low-temperature rheological properties by DMP-30. Therefore, in practical engineering applications, when this WEREA is used in cold regions with low temperatures below −12°C, additional measures (such as adjusting the ratio of curing agent or adding low-temperature modifiers) should be taken to improve its low-temperature creep performance, ensuring the long-term stability and durability of the pavement.

#### 3.2.3. Adhesive properties.

**Fig 8:**
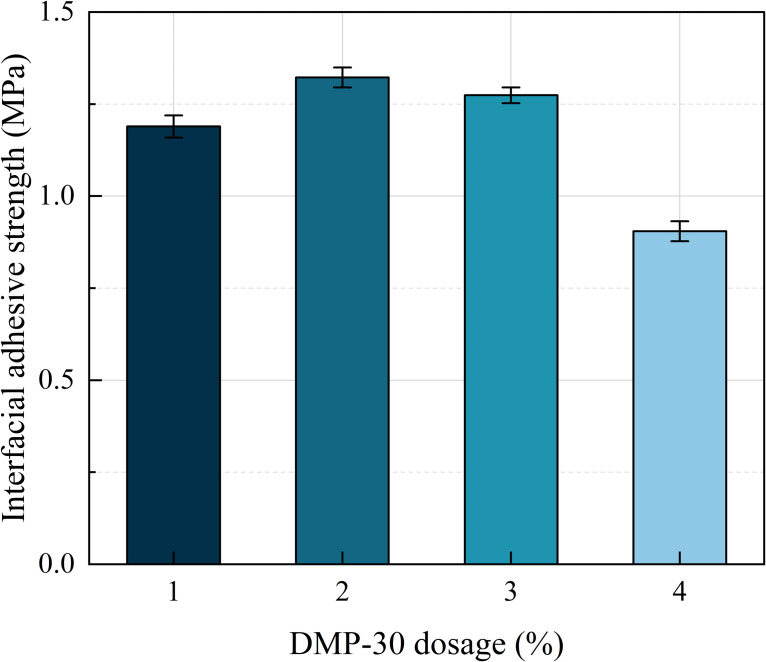
Adhesive strength of WEREA at various DMP-30 dosages.

Fig 8 shows the interfacial adhesive strength of the samples at various DMP-30 dosages. As the dosage of DMP-30 increases, the interfacial adhesive strength initially rises and then declines. The maximum adhesive strength is achieved at a 1% dosage of DMP-30, indicating that an appropriate amount of DMP-30 contributes to synthesizing WEREA with optimal adhesive properties. Moreover, DMP-30 has both positive and negative effects on the adhesion performance of WEREA. Dosages of 1% and 2% exhibit improvement effects, while a dosage of 3% shows a weakening effect. This may be due to the fact that DMP-30, as a more active amine, can participate in the curing reaction of epoxy resin. Excessive amounts of DMP-30 lead to increased consumption of epoxy resin, thereby reducing the adhesion performance between the curing agent and epoxy resin [22].

### 3.3. Microscopic properties(FTIR analysis)

**Fig 9:**
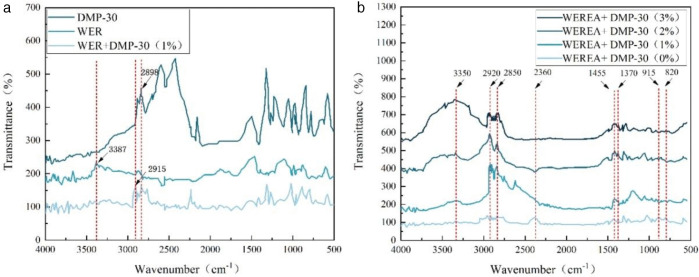
The results of Fourier transform infrared spectroscopy test. **(a)**WER + DMP-30. **(b)** WEREA+DMP-30.

As can be seen in Fig 9(a), the characteristic peaks of DMP-30 and WER + DMP-30 at 2915 cm^-1^ and 2898 cm^-1^ may be due to the vibration of CH_3_ [12,36]. The characteristic peak of WER near 3387 cm^-1^ may be due to the N-H vibration in the curing agent [5,14]. Compared to WER, the N-H stretching vibration peak near 3387 cm^-1^ of WER + DMP-30 (1%) is significantly reduced, suggesting a reaction between the curing agent and the epoxy resin promoted by DMP-30.

From Fig 9(b), it can be seen that the stretching vibration peaks of CH_2_ existed at 2920 cm^-1^ and 2852 cm^-1^, and the variable angle vibration peaks of CH_3_ existed at 1455 cm^-1^ and 1370 cm^-1^ for each sample, which are characteristic peaks commonly found in asphalt [37]. The epoxy group vibrational expansion peaks near 915 cm^-1^ and 820 cm^-1^ gradually disappear after the addition of DMP-30, which indicates that the addition of DMP-30 promotes the curing reaction of WER. Furthermore, there is an increase in N-H shrinkage vibration peak near 3350 cm^-1^with an increment in DMP-30 dosage. The peak here can reflect the amount of remaining curing agent, which may be due to the fact that DMP-30 promotes the reaction of amine curing agent with WER and also reacts with WER, resulting in the remaining amine curing agent.

### 3.4. Effect of DMP-30 on waterborne epoxy micro-surface mixture

#### 3.4.1. High temperature stability.

**Fig 10:**
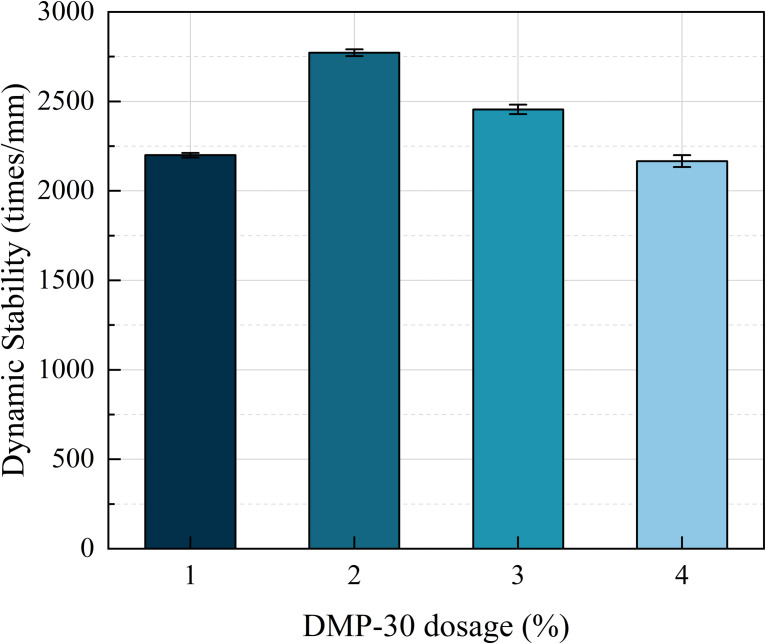
Dynamic stability of mixtures at micro-surface with various DMP-30 dosages.

The dynamic stability of the samples with various dosages of DMP-30 is illustrated in Fig 10. As depicted, an increase in DMP-30 dosage initially enhances the dynamic stability of the samples, followed by a subsequent decline. When the DMP-30 dosage is 1%, the dynamic stability of the sample reaches the maximum value of 2765 times/mm. This observation indicates that an optimal amount of DMP-30 (1%) can improve the high temperature stability of the samples to a certain extent.

#### 3.4.2. Freeze-thaw stability.

**Fig 11:**
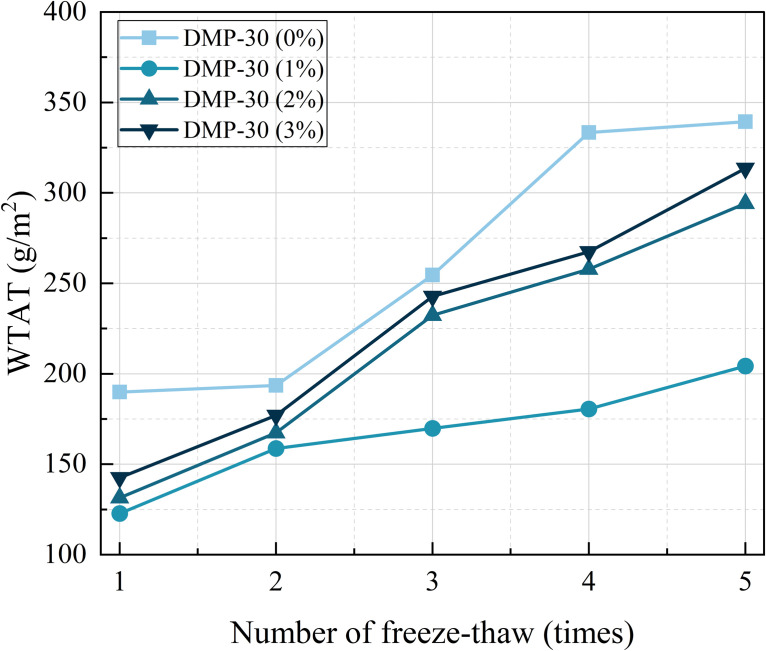
Freeze-thaw WTAT of samples at various DMP-30 dosages.

The Freeze-thaw WTAT of the samples with various DMP-30 dosages is illustrated in Fig 11. It can be observed from the figure that, under the same DMP-30 dosage, the WTAT of the samples exhibits an increasing trend as the number of freeze-thaw cycles increases. This may be attributed to the fact that WER is a thermosetting material, which becomes more brittle at low temperatures after molding on micro-surfaces. Previous studies have indicated that the increased brittleness of the resin matrix at low temperatures makes it susceptible to the initiation and propagation of microcracks under the stress of freeze-thaw cycles. Consequently, the freeze-thaw cycle process destroys the structural strength of the sample [38]. Combined with the results of the low-temperature binder performance analysis, the admixture of DMP-30 may inhibit this phenomenon. Taking three freeze-thaw cycles as an example, compared with DMP-30 (0%), the Freeze-thaw WTAT of samples decreases by 33.28%, 8.45%, and 4.52% when the dosage of DMP increased from 1% to 3%. Among them, samples with DMP-30 (1%) exhibited the most significant decrease in WTAT, indicating that DMP-30 (1%) WEREA micro-surface provides superior freeze-thaw stability.

#### 3.4.3. Water stability.

**Fig 12:**
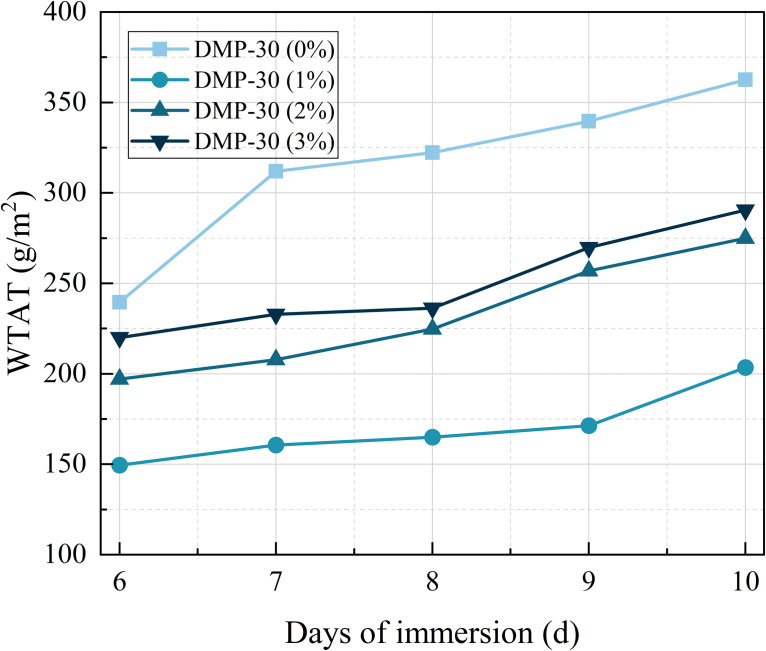
Immersion WTAT of samples at various DMP-30 dosages.

The immersion WTAT of the samples at various DMP-30 dosages is illustrated in Fig 12. It can be observed from the figure that, when the dosage of DMP-30 remains constant, the WTAT of the samples exhibits an increasing trend with the increase in immersion days. When the DMP-30 dosage increased from 1% to 3%, the WTAT of the samples decreased by 48.70%, 30.19%, and 26.66%, respectively, compared with DMP-30 (0%). The DMP-30 (1%) sample has the best water stability. As the dosage increases, the water stability of the sample becomes worse. This may be due to the excessive chemical reaction of DMP-30 with WER, which affects the structural strength of the micro-surface [16].

#### 3.4.4. Curing conditions.

The results of MD simulations demonstrate that the diffusion rate of the components in the WER emulsion system is influenced by temperature, which subsequently affects the curing reaction rate and strength formation of binders and mixes. To eliminate the influence of moisture on micro-surface strength molding, this study conducts dry track abrasion tests at temperatures of 15°C, 25°C, and 35°C. The test results are presented in Table 7.

**Table 7:** Abrasion test results at different curing temperatures, DMP-30 dosages, and curing times.

Temperature (℃)	DMP-30 dosage (%)	Abrasion value (g/m^2^)						
		8h	10h	12h	14h	16h	18h	20h
15	0	–	1598.6	969.0	650.9	286.0	295.0	301.0
	1	–	785.0	573.0	386.0	162.0	143.0	135.0
	2	–	860.2	623.5	415.2	186.1	203.4	185.7
	3	–	1013.1	755.1	535.2	225.1	233.6	231.1
25	0	1232.2	986.0	812.0	468.0	236.0	254.0	253.0
	1	812.0	725.0	420.0	162.0	130.0	125.0	135.0
	2	886.2	780.2	560.1	330.1	230.2	170.2	165.2
	3	986.3	830.6	612.1	365.1	256.2	221.1	212.3
35	0	953.0	695.0	495.0	205.0	165.0	172.0	176.0
	1	637.0	397.0	265.1	72.0	80.0	75.2	76.0
	2	705.3	406.2	303.1	112.1	96.2	88.2	91.4
	3	768.2	445.1	342.1	135.3	136.2	129.6	142.8

From the table, at the same DMP-30 dosage, the temperature was increased from 15°C to 35°C, and the abrasion values of the specimens showed a decreasing trend. Taking DMP-30 (1%) as an example, the abrasion value is reduced by 53.8% after 12h of curing, which indicates that temperature plays a significant role in WEREA curing as well as the strength formation of the mixture. This phenomenon can be attributed to the thermodynamic acceleration effect: an increase in temperature inherently increases the diffusion rate of the epoxy resin, curing agent, and DMP-30, leading to a more thorough mixing of molecules and, consequently, a more complete curing reaction. The macroscopic abrasion data indicates that each 10°C increase significantly enhances the effective curing rate, allowing for an extension of the construction window in temperate climates.

In addition, DMP-30 can reduce the abrasion loss of specimens at lower temperatures. Taking 12h of curing as an example, the abrasion value of 15°C and DMP-30 (1%) is 573 g/m^2^, which is much smaller than the abrasion loss value of 25°C and DMP-30 (0%) of 812 g/m^2^, which can be explained from the perspective of chemical kinetics: DMP-30 maintains high reactivity across different temperatures, and its introduction promotes a more complete reaction between the epoxy resin and the curing agent even at lower temperatures. This effectively achieves the same reduction in abrasion loss as would be expected from a higher ambient temperature. In a word, the experimental results in Table 7 can also provide guidance on the conditioning of waterborne epoxy-emulsified asphalt micro-surface mixtures. In practical engineering applications, to ensure the early-stage strength and abrasion resistance of microsurfacing mixtures, it is recommended to consistently incorporate 1% DMP-30 curing accelerator. This dosage significantly reduces abrasion values across a range of temperature conditions. Particularly in low-temperature environments, it effectively shortens the curing period—which would otherwise be an extremely long and indeterminate duration—to within a standard construction cycle (approximately 16 hours), thereby offering significant practical value in engineering practice.

The abrasion resistance of WEREA micro-surfacing is essentially determined by the surface hardness and internal cohesion of the binder, which are closely related to the degree of curing reaction. DMP-30, as a curing accelerator, promotes the cross-linking reaction between WER and curing agent by increasing the molecular diffusion rate, thereby forming a denser and more uniform three-dimensional network structure in the binder. This dense network structure significantly improves the surface hardness of WEREA.

## 4. Conclusions

The primary objective of this study is to explore the impact of DMP-30 curing accelerator on the properties of WEREA and its micro-surface mixes, supported by FTIR to provide microscopic information. Finally, a recommended dosage of DMP-30 is proposed. The main conclusions drawn are as follows:

(1) A 15% dosage of WER is acceptable. Beyond this threshold, the internal WEREA will form a complete three-dimensional mesh structure, resulting in deceleration of the increase in adhesion strength. This suggests that 15% is the optimal dosage balancing economic costs and mechanical performance.
(2) The DMP-30 has both positive and negative effects on the high-temperature and adhesion properties of WEREA. The G*/sinδ and adhesion strength of specimens are the maximum at a 1% DMP-30 dosage. As the DMP-30 dosage rises, the enhancement effect gradually diminishes, and the negative effect becomes apparent at a 3% dosage. This phenomenon can be explained by the competition between excessive DMP-30 and the curing agent for epoxy groups, which disrupts the stoichiometric balance.
(3) FTIR test results indicate that a moderate amount (1%) of DMP-30 primarily facilitates the reaction between epoxy resin and curing agent. With rising DMP-30 dosage, the N-H characteristic peak near 3350 cm-1 significantly increases, suggesting that excessive DMP-30 itself may react with the epoxy resin, resulting in a large amount of residual curing agent. This microscopic evidence confirms that stoichiometric imbalance caused by excessive accelerator is the fundamental reason for the deterioration of macroscopic performance.
(4) DMP-30 can enhance the high temperature stability, freeze-thaw stability, and water stability of WEREA micro-surfaces. As the DMP-30 dosage increases from 0% to 3%, the dynamic stability exhibits a trend of initially increasing and then decreasing. By contrast, the freeze-thaw and immersion WTAT show the opposite trend, with the best performance achieved at 1%. Furthermore, abrasion test results indicate that the incorporation of 1% DMP-30 at higher temperatures can minimize the abrasion loss in a shorter period of time. The synergistic effect of DMP-30 and higher curing temperatures offers a viable strategy for extending the construction window for micro-surface treatments in temperate climates.

Admittedly, this study has certain limitations. While macroscopic abrasion tests demonstrated the temperature-dependent curing behavior of the WEREA system, atomic-scale diffusion kinetics and intermolecular interactions were not quantitatively captured. Future work will utilize Fluorescence Microscopy or Confocal Raman Microscopy to provide direct visual and spectral evidence of phase separation and diffusion processes, validating the macroscopic observations.

## References

[1] Han Y, Wang Z, Zhao S, Wang J. AC impedance function of electrochemical working station as novel curing degree monitor method: A model curing system of epoxy/anhydride/DMP-30. Measurement. 2019;145:600–10. doi: 10.1016/j.measurement.2019.05.103

[2] Luo X, Wang F, Gong H, Tao J, Qiu X, Wang N. Effectiveness Evaluation of Preventive Maintenance Treatments on Asphalt Pavement Performance Using LTPP Data. Int J Pavement Res Technol. 2021;15(5):1139–54. doi: 10.1007/s42947-021-00078-2

[3] Luo Y, Zhang K, Xie X, Yao X. Performance evaluation and material optimization of Micro-surfacing based on cracking and rutting resistance. Constr Build Mater. 2019;206:193–200. doi: 10.1016/j.conbuildmat.2019.02.066

[4] Cai X, Huang W, Liang J, Wu K. Study of Pavement Performance of Thin-Coat Waterborne Epoxy Emulsified Asphalt Mixture. Front Mater. 2020;7:88. doi: 10.3389/fmats.2020.00088

[5] He L, Hou Y, Yang F. Study of the Properties of Waterborne Epoxy Resin Emulsified Asphalt and Its Modification Mechanism. J Mater Civ Eng. 2023;35(6). doi: 10.1061/jmcee7.mteng-14627

[6] Ji X, Gao L, Zhang Y, Gao J. An investigation on the compatibility of epoxy resin and asphalt based on molecular simulation. J Phys Conf Ser. 2023;012005.

[7] Li M, Min Z, Wang Q, Huang W, Shi Z. Effect of epoxy resin content and conversion rate on the compatibility and component distribution of epoxy asphalt: A MD simulation study. Constr Build Mater. 2022;319:126050. doi: 10.1016/j.conbuildmat.2021.126050

[8] Liu Q, Ding G, Zhang Z, Fu C, Oeser M. Investigation on bitumen-epoxy interface in cold mixed epoxy bitumen using experimental observation and molecular dynamics simulation. Constr Build Mater. 2021;303:124490. doi: 10.1016/j.conbuildmat.2021.124490

[9] Yip P, Lee YG, Park TS. Analysis of waterborne epoxy modified emulsified asphalt properties & characteristics. J Korean Asphalt Instit. 2020;10(1):27–36.

[10] Guo X, Li Y, Zhai R, Sun B, Solaimanian M. The curing time effect on SBS-modified asphalt performance: a combined molecular dynamics and mechanical testing study. Mater Res Express. 2025;12(10):105301. doi: 10.1088/2053-1591/ae1284

[11] Zhang W, Guo X, Wang K, Zhao L, Yao H, Zhai R, et al. Study on the Effects and Mechanisms of Different RPE / RPP Ratios on the Properties of Modified Asphalt. J Appl Polymer Sci. 2025;143(4). doi: 10.1002/app.58114

[12] Li R, Leng Z, Zhang Y, Ma X. Preparation and characterization of waterborne epoxy modified bitumen emulsion as a potential high-performance cold binder. J Clean Product. 2019;235:1265–75. doi: 10.1016/j.jclepro.2019.06.267

[13] He Y, Zheng N, Xu A. Modification Mechanism and Adhesive Performance of Waterborne Epoxy Resin-SBR Composite Modified Emulsified Asphalt. In: CICTP 2020. 2020. p. 1705–14.

[14] Liu F, Zheng M, Fan X, Li H, Wang F, Lin X. Properties and mechanism of waterborne epoxy resin-SBR composite modified emulsified asphalt. Constr Build Mater. 2021;274:122059. doi: 10.1016/j.conbuildmat.2020.122059

[15] Liu M, Han S, Wang Z, Ren W, Li W. Performance evaluation of new waterborne epoxy resin modified emulsified asphalt micro-surfacing. Constr Build Mater. 2019;214:93–100. doi: 10.1016/j.conbuildmat.2019.04.107

[16] Li R, Leng Z, Partl MN, Raab C. Characterization and modelling of creep and recovery behaviour of waterborne epoxy resin modified bitumen emulsion. Mater Struct. 2021;54(1). doi: 10.1617/s11527-020-01594-6

[17] Zhang Z, Wang S, Lu G. Properties of new cold patch asphalt liquid and mixture modified with waterborne epoxy resin. Int J Pavement Eng. 2019;21(13):1606–16. doi: 10.1080/10298436.2018.1559314

[18] Zhang YJ, Wu WM, Cao HS, Zhao LD, Zhang YL, Liu BQ, et al. Investigation and Evaluation of the Emulsified Asphalt with Waterborne Epoxy Resin. KEM. 2020;842:337–45. doi: 10.4028/www.scientific.net/kem.842.337

[19] Chen W, Zheng M, Wang H. Evaluating the Tire/Pavement Noise and Surface Texture of Low-Noise Micro-Surface Using 3D Digital Image Technology. Front Mater. 2021;8. doi: 10.3389/fmats.2021.683947

[20] Gu Y, Tang B, He L, Yang F, Wang H, Ling J. Compatibility of cured phase-inversion waterborne epoxy resin emulsified asphalt. Constr Build Mater. 2019;229:116942. doi: 10.1016/j.conbuildmat.2019.116942

[21] Chen T, Gu GF. Study on novel waterborne epoxy resin emulsion and its film formation. J Build Mater. 2001;4(4):356–61.

[22] Hui B, Li Y, Zhang YD, Yang XY. Regulation Efficiency and Mechanism of Curing-Demulsification Ｒate of Waterborne Epoxy Emulsified Asphalt. Mater Rep. 2022;36(16):22050008–6.

[23] Lv J, Huang J, Wu H, Zhang Y, Qiu J, Sun X, et al. Experience Study on Long‐Life Microsurfacing with High Water Resistance Performance. Adv Mater Sci Eng. 2021;2021(1). doi: 10.1155/2021/2487478

[24] Xu W, Shah YI, Cheng Z, Li Y, Zhang K, Yu X. Laboratory design and evaluation of a functional color micro-surfacing with synthetic resin-based binder. Road Mater Pavement Des. 2022;24(10):2343–62. doi: 10.1080/14680629.2022.2141134

[25] Han S, Yao T, Han X, Hongwei Z, Yang X. Performance evaluation of waterborne epoxy resin modified hydrophobic emulsified asphalt micro-surfacing mixture. Constr Build Mater. 2020;249:118835. doi: 10.1016/j.conbuildmat.2020.118835

[26] Ji J, Liu LH, Suo Z, Xu Y, Yang S, Xu SF. Performances of micro surfacing with waterborne epoxy resin modified emulsified asphalt. J Chang'an Univ (Nat Sci Ed). 2017;37(5):23–30.

[27] Chen Y, Hossiney N, Yang X, Wang H, You Z. Application of Epoxy‐Asphalt Composite in Asphalt Paving Industry: A Review with Emphasis on Physicochemical Properties and Pavement Performances. Adv Mater Sci Eng. 2021;2021(1). doi: 10.1155/2021/3454029

[28] Guo X. A Data‐Driven Framework for Explainable Artificial Intelligence in Pavement Distress Analysis and Decision Support: Integrating Clustering Models and Principal Component Analysis. Struct Control Health Monit. 2025;2025(1). doi: 10.1155/stc/8852297

[29] Guo X, Wang N, Li Y. Enhancing pavement maintenance: A deep learning model for accurate prediction and early detection of pavement structural damage. Constr Build Mater. 2023;409:133970. doi: 10.1016/j.conbuildmat.2023.133970

[30] Guo X, Wang N. Automated Identification of Pavement Structural Distress Using State-of-the-Art Object Detection Models and Nondestructive Testing. J Comput Civ Eng. 2024;38(4). doi: 10.1061/jccee5.cpeng-5864

[31] Li Y, Guo X, Wang X, Wang X, Xu J, Wang C, et al. Investigation on the survival and activating behavior of rejuvenator-loaded fibers in asphalt pavement. Constr Build Mater. 2025;475:141282. doi: 10.1016/j.conbuildmat.2025.141282

[32] Ai T, Pang H, Wu X, Zhong D, Yang K, Yan X, et al. Preparation and Properties of Waterborne Epoxy-Resin-Emulsified Asphalt Modified by Oxidized Extraction Oil. Buildings. 2022;12(12):2133. doi: 10.3390/buildings12122133

[33] Yang G, Wang C, Fu H, Yan Z, Yin W. Waterborne Epoxy Resin–Polyurethane–Emulsified Asphalt: Preparation and Properties. J Mater Civ Eng. 2019;31(11). doi: 10.1061/(asce)mt.1943-5533.0002904

[34] Sun Y, Chen L, Cui L, Zhang Y, Du X. Molecular dynamics simulation of cross-linked epoxy resin and its interaction energy with graphene under two typical force fields. Comput Mater Sci. 2018;143:240–7. doi: 10.1016/j.commatsci.2017.11.007

[35] Martínez-Anzures JD, Zapién-Castillo S, Salazar-Cruz BA, Rivera-Armenta JL, Antonio-Cruz R d C, Hernández-Zamora G, et al. Preparation and properties of modified asphalt using branch SBS/nanoclay nanocomposite as a modifier. Road Mater Pavement Des. 2019;20(6):1275–90.

[36] Kezhen Y, Junyi S, Kaixin S, Min W, Goukai L, Zhe H. Effects of the chemical structure of curing agents on rheological properties and microstructure of WER emulsified asphalt. Constr Build Mater. 2022;347:128531. doi: 10.1016/j.conbuildmat.2022.128531

[37] Wang D, Hu L, Dong S, You Z, Zhang Q, Hu S, et al. Assessment of testing methods for higher temperature performance of emulsified asphalt. J Clean Production. 2022;375:134101. doi: 10.1016/j.jclepro.2022.134101

[38] Chen Q, Wang C, Yu S, Song Z, Fu H, An T. Low-temperature mechanical properties of polyurethane-modified waterborne epoxy resin for pavement coating. Int J Pavement Engineering. 2022;24(2). doi: 10.1080/10298436.2022.2099853

